# Acute pancreatitis or severe increase in pancreatic enzyme levels following remdesivir administration in COVID-19 patients: an observational study

**DOI:** 10.1038/s41598-022-09170-4

**Published:** 2022-03-29

**Authors:** Kazuhito Miyazaki, Yukihiro Yoshimura, Nobuyuki Miyata, Hiroaki Sasaki, Aya Shiba, Masaharu Aga, Yusuke Hamakawa, Yuri Taniguchi, Yuki Misumi, Yoko Agemi, Tsuneo Shimokawa, Hiroaki Okamoto, Natsuo Tachikawa

**Affiliations:** 1 Department of Respiratory Medicine, Yokohama Municipal Citizen’s Hospital, Yokohama, Kanagawa Japan; 2 Departments of Infectious Diseases, Yokohama Municipal Citizen’s Hospital, Yokohama, Kanagawa Japan

**Keywords:** Viral infection, Respiratory tract diseases

## Abstract

Remdesivir has been shown to reduce recovery time and mortality among patients with coronavirus disease 2019 (COVID-19). However, data regarding the efficacy and safety of remdesivir use are limited in Japan. We conducted a single-center retrospective cohort study at Yokohama Municipal Citizen’s Hospital, Kanagawa, Japan. Patients with COVID-19 pneumonia treated with remdesivir were included. The onset of acute pancreatitis and increased pancreatic enzyme levels and clinical, laboratory, treatment, and outcome data were collected and analyzed. A total of 201 patients were included. Among the 201 patients treated with remdesivir, 177 recovered from COVID-19. Increased pancreatic enzyme levels of grade 3 or higher or acute pancreatitis developed in 23 of the 201 patients. The potential etiopathogenetic effects of remdesivir on increased pancreatic enzyme levels of grade 3 or higher or acute pancreatitis were ascertained by reviewing the characteristics of patients hospitalized for COVID-19 who did not receive remdesivir treatment. Only 3 of 159 patients had increased pancreatic enzyme levels of grade 3 or higher during the treatment course. Multivariate analysis indicated remdesivir administration and severe COVID-19 infection by National Institute of Health standards as independent risk factors. Acute pancreatitis and severe increases in pancreatic enzyme levels were observed among patients with COVID-19 treated with remdesivir.

## Introduction

The coronavirus disease 2019 (COVID-19) pandemic caused by severe acute respiratory syndrome coronavirus-2 (SARS-CoV-2) remains a global health burden. As of August 2021, more than 206 million people have been infected with SARS-CoV-2 and more than 4.3 million people have succumbed to COVID-19^1^. Remdesivir is an antiviral agent that inhibits viral RNA-dependent RNA polymerase and was originally developed for treating Ebola virus infection; however, remdesivir has been found to be effective against SARS-CoV-2^2,3^. In the Adaptive COVID-19 Treatment Trial (ACTT-1), remdesivir reduced the median recovery time from 15 (placebo group) to 10 days. Moreover, remdesivir reduced mortality rate from 11.9% (placebo group) to 6.7% on day 15 and from 15.2% (placebo group) to 11.4% on day 29 after hospitalization^4,5^. The United States Food and Drug Administration approved the drug for emergency use (Emergency Use Authorization) in patients with severe COVID-19 on May 1, 2020, and in Japan, remdesivir was granted special approval on May 7, 2020, under Japan’s Pharmaceutical and Medical Devices Act to permit its urgent use as a treatment option for infections caused by SARS-CoV-2. Only a limited number of people have been administered remdesivir for COVID-19 in Japan, and there are limited data on the efficacy and safety of remdesivir. Thus, studies on the adverse effects of remdesivir are needed.

As of January 2021, remdesivir was administered to 201 patients with COVID-19 pneumonia in our hospital in Kanagawa, Japan. Since the outbreak on the Diamond Princess cruise ship in February 2020, our hospital has been using steroids to treat respiratory failure in COVID-19 patients because we lack the facilities to perform extracorporeal membrane oxygenation. Thus, most patients who were administered remdesivir were also administered steroids. All but 22 patients survived, and therapy was equally as efficacious or better than previously reported. However, unlike previous COVID-19 studies^4–8^, the frequency of increased levels of pancreatic enzymes and pancreatitis was 11.4% (23/201) in this study. The pancreatitis was mild and resolved after supportive therapy. However, pancreatitis is a serious adverse event that can be potentially life-threatening if left untreated.

The aim of this study was to assess the association between remdesivir administration and elevated pancreatic enzymes in COVID-19 patients. A secondary aim was to report the efficacy and other adverse events observed in patients treated with remdesivir in a Japanese hospital setting.

## Methods

### Patients selection

This single-center retrospective observational study was conducted in the Department of Infectious Diseases, Yokohama Municipal Citizen’s Hospital, Kanagawa, Japan. In total, 201 hospitalized adult patients with COVID-19 (aged ≥ 20 years) who were administered remdesivir during the time period from June 1, 2020, to January 31, 2021, were included in the study. As a control group for remdesivir administration, adult patients with COVID-19 who were hospitalized at our hospital but were not administered remdesivir in the time period between February 5, 2020, and January 31, 2021 were enrolled. Patients enrolled in other trials, those without laboratory results of pancreatic enzyme levels, those transferred to our hospital after administration of remdesivir at another hospital and did not receive remdesivir at our hospital, or those who contracted cluster infections at other hospitals during the onset of acute pancreatitis were excluded. Patients who refused to participate in the study were excluded. The data cutoff date was March 20, 2021.

### Severity classification

The National Institutes of Health (NIH) Clinical Presentation of People with SARS-CoV-2 Infection guidelines were used to classify the severity of COVID-19^9^.

### Adverse events

Adverse events because of remdesivir administration were evaluated using the Common Terminology Criteria for Adverse Events version 5.0 (CTCAE ver 5.0).

### Data collection

This was a retrospective observational study, and, despite the frequency of blood collection being determined by the attending physician, blood samples were generally collected 2–3 times per week. Blood samples were collected for biochemical analysis, including amylase and lipase, blood counts, and coagulation. Adverse events of grade 1 or higher on the CTCAE ver 5.0 were collected as much as possible in the remdesivir group, and only necessary items were collected in the control group.

### Statistical analysis

The association between a grade 3 or higher increase in pancreatic enzyme levels or acute pancreatitis and patient characteristics was analyzed using the chi-square test. Statistical significance was set at *p* ≤ 0.05. A logistic regression model was used to calculate the adjusted odds ratio (OR) with 95% confidence interval (CI) for the risk of increase in grade 3 or higher pancreatic enzyme levels or the onset of acute pancreatitis. We used multivariable logistic regression to control for potentially confounding roles of remdesivir administration, steroid administration, sex, age, severity classification, comorbidities (diabetes, hypertension, hyperlipidemia), and D-dimer level elevation. These analyses were performed using the Statistical Package for Social Science (SPSS) version 23 (IBM Corp., Armonk, NY). Missing data on diabetes were considered not examined, and at the time of analysis, analysis was performed with diabetes versus without diabetes or not examined.

### IRB approval

The study protocol was approved by the independent ethics committee of the Institutional Review Board of Yokohama Municipal Citizen’s Hospital (No. 20-12-01). The study was a non-interventional retrospective observational study, and the ethics committee of the institutional review board approved the opt-out method of consent without written consent. Informed consent was obtained in the form of an opt-out notice displayed on the institutional website. Patients who refused to participate in the study were excluded.

### Statement of human rights

All procedures performed in this study involving human participants were in accordance with the ethical standards of the institutional and/or national research committee and with the 1964 Helsinki declaration and its later amendments or comparable ethical standards.

## Results

### Patients characteristics

**Figure 1:**
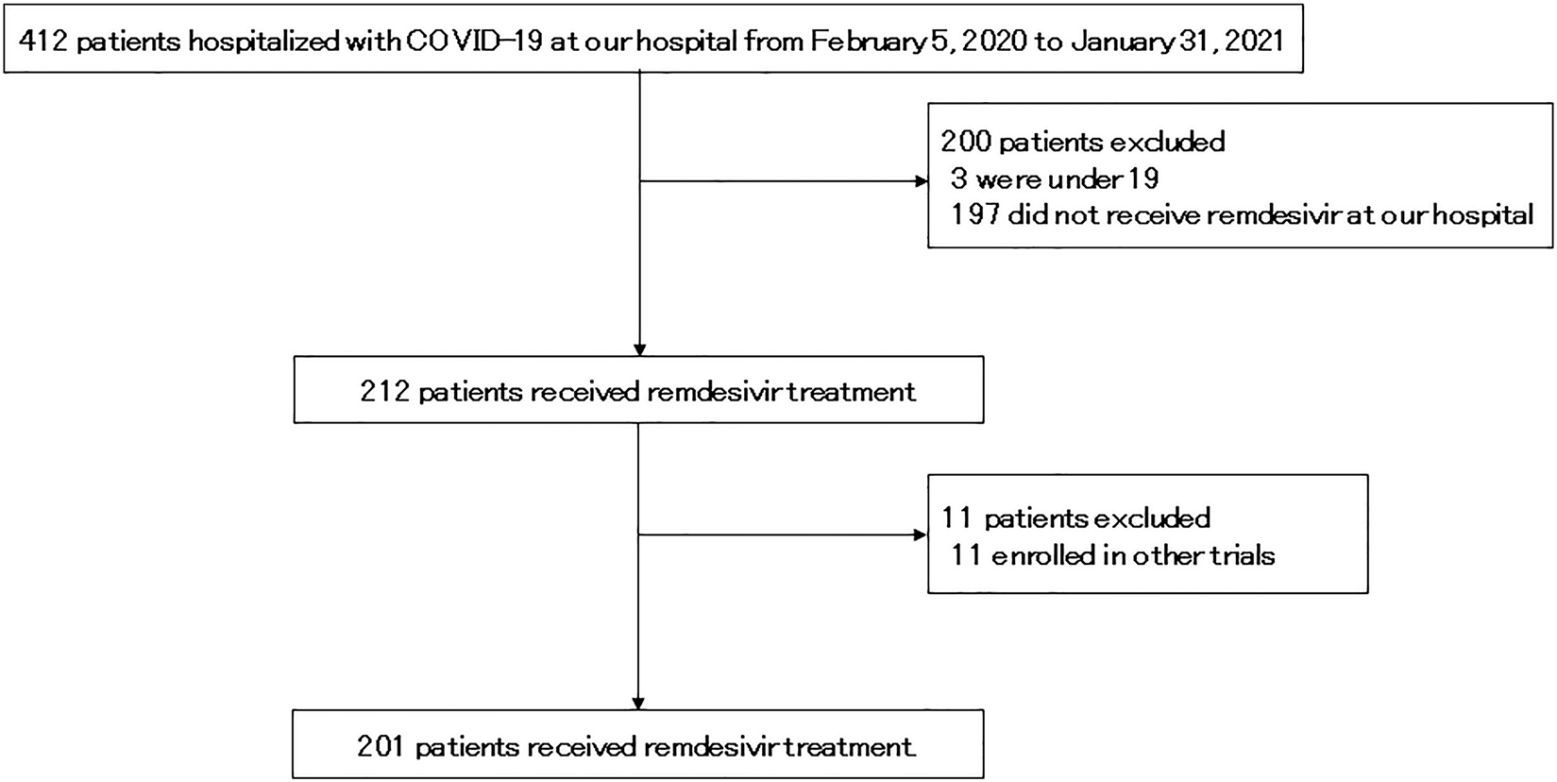
The diagram of patient selection.

The baseline patient characteristics are shown in Table 1. Between June 2020 and January 2021, 201 patients who received remdesivir were enrolled in this study (Fig. 1). The median age was 73 (range: 22–98) years; 125 patients (61.2%) were male, and 194 patients were Japanese. Based on the COVID-19 severity classification developed by the NIH^9^, 2, 151, and 58 cases were moderately, severely, and critically ill, respectively. Among the participants, 81 were never-smokers; 64 were ex-smokers; 19 were current smokers, and 37 were unknown. Moreover, 96.0% (193/201) of patients were administered steroids; 60 patients received pulse steroid therapy (methylprednisolone, 1000 mg/day) and 133 were receiving non-pulse steroid therapy. The median duration of remdesivir administration was 5 days (range, 1–10 days). The median duration of steroid administration was 10 days (range, 2–69 days). In total, 111 adverse events classified as grade 3 or higher were observed in 69 patients (Table 2). At the time of analysis, 177 patients were discharged, 22 died, and two were still hospitalized. The mortality rate was 10.9%, and the median duration of hospitalization was 14 days (range, 1–89 days). Under these conditions, the mortality rate was 10.9%, which is comparable to the results of the ACTT-1 trial.

**Table 1:** Characteristics of COVID-19 patients who received remdesivir.

Patients characteristics (N = 201)	N	(%)
Age, years, median (range)	73 (22–98)	
**Sex**		
Male	125	61.2
Female	76	37.8
**Race**		
Japanese	194	96.5
East-Asian	6	3.0
Lationo	1	0.5
**Smoking history**		
Never smoker	81	40.3
Ex-smoker	64	31.8
Current smoker	19	9.5
Unknown	37	18.4
**History of alcohol consumption**		
Regular drinking	52	25.9
Drinking on occasion	28	14.0
Past drinker	5	2.5
Never	59	29.4
Unknown	57	28.4
**Comorbidities**		
Diabetes mellitus	67	33.3
Hypertension	109	54.2
Hyperlipidemia	40	20.0
**D-dimer, µg/mL**		
Normal	28	13.9
Elevated (> 1.0 μg/mL)	172	85.6
Unexamined	1	0.5
**Severity of illness categories by NIH classification**		
Moderate	2	1.0
Severe	151	75.1
Critical	58	28.9
**Duration of remdesivir administration, days**		
1	2	1.0
2	4	2.0
3	3	1.5
4	2	1.0
5	173	86.1
7	1	0.5
8	1	0.5
10	15	7.5
Duration from COVID-19 onset to remdesivir administration, days, median (range)	6 (0–14)	
**Steroid dose**		
Steroid pulse	60	29.9
Non-pulse	133	66.2
No	8	4.0
Median days of steroid administration	10 (2–69)	
**Outcome**		
Discharged	177	88.1
Death	22	10.9
Hospitalized	2	1.0
Duration of hospitalization, days, median (range)	14 (1–89)	

Baseline demographic and clinical characteristics of 201 patients who received remdesivir between J June 1, 2020 to January 31, 2021.

**Table 2:** Grade 3 or more adverse events in COVID-19 patients who received remdesivir treatment.

	Grade 3	Grade 4	Grade 5
Infection	17	8	2
Septic shock	0	3	0
Aspiration	4	1	0
Hyperkalepia	2	1	0
Hypokalemia	0	1	0
Hypernatremia	0	1	0
Hyponatremia	0	1	0
Aspartate aminotransferase increase	1	1	0
Alanine aminotransferase increase	2	1	0
γ-Glutamyltransferase increase	2	0	0
Blood bilirubin increase	1	0	0
Alkaline phosphatase increase	1	0	0
Serum amylase increase	3	3	0
Lipase increase	16	6	0
Pancreatitis	3	0	0
Pneumonitis	1	1	0
Pneumothorax	2	0	0
Bleeding	1	0	0
Upper gastrointestinal hemorrhage	1	0	0
Lower gastrointestinal hemorrhage	1	0	0
Gastric ulcer	1	0	0
Diarrhea	1	0	0
Acute kidney injury	3	0	0
Stroke	1	0	0
Thromboembolic event	1	0	0
Rash maculo-papular	1	0	0
Shingles	1	0	0
Delirium	11	1	0
Psychosis	1	1	0

Among the adverse events (AEs) assessed using CTCAE version 5, AEs of Grade 3 or higher are described.

### Acute pancreatitis or severe elevation of pancreatic enzyme levels after remdesivir treatment

**Figure 2:**
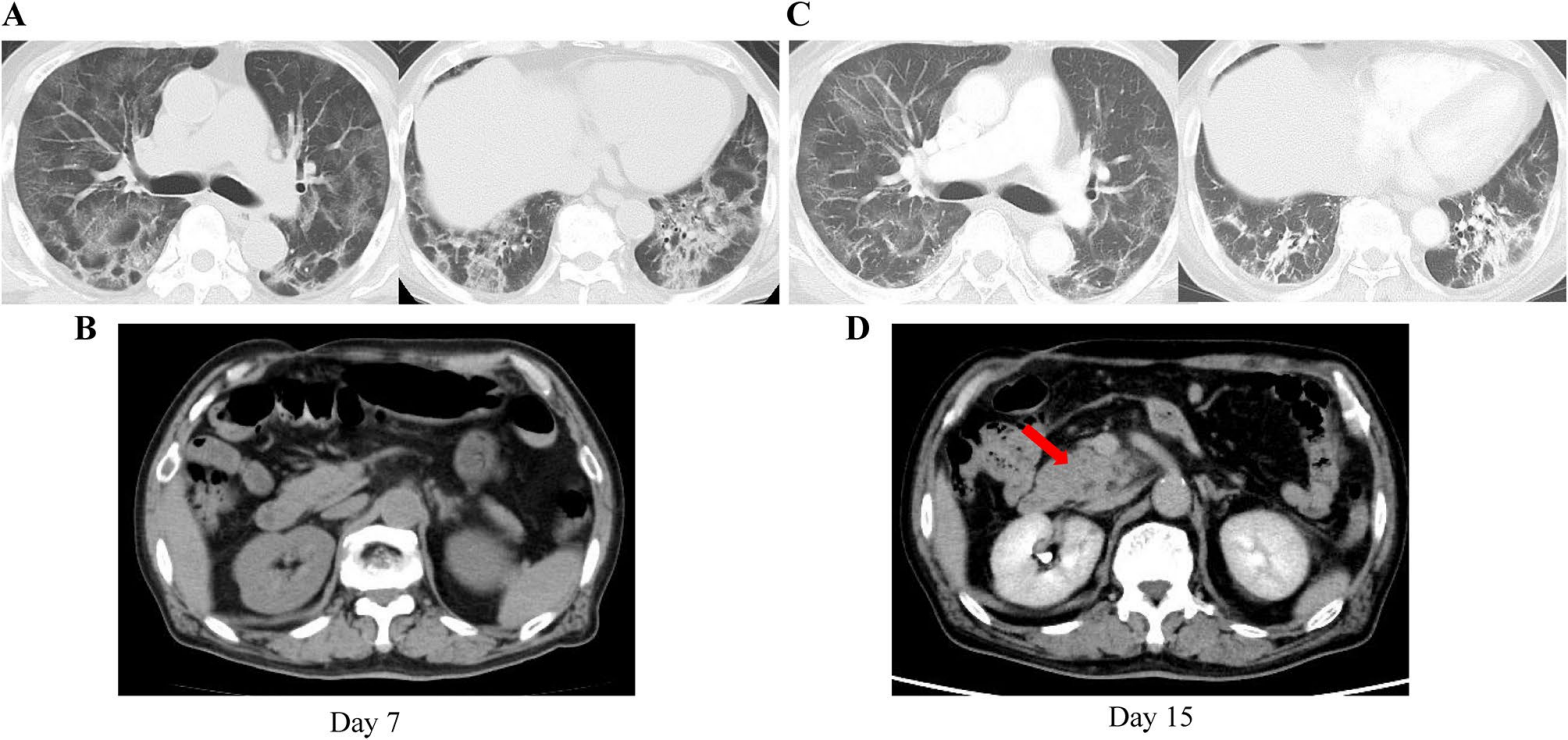
Computed tomography (CT) scan of a patient who developed acute pancreatitis after remdesivir administration. This is one of the three cases of acute pancreatitis. CT was performed at the time of admission to our hospital (7 days after COVID-19 onset) and 15 days after COVID-19 onset, when an increase in pancreatic enzyme levels was observed. (**A**) During hospitalization, extensive ground-glass shadows were observed in the bilateral lung fields. (**B**) Abdominal CT on day 7. (**C**) However, the ground-glass shadows dissipated slightly by day 15, showing some contractile changes. (**D**) Contrast-enhanced CT image was obtained when pancreatic enzyme levels were elevated, and it shows an enlarged uncinate process of the pancreas and an increase in the concentration of surrounding fatty tissue, which were not observed at the time of admission (**B**).

A grade 3 or higher increase in pancreatic enzyme levels was observed in 23 of 201 patients (Table 3). The median age was 73 (range: 50–91) years; 14 patients (60.9%) were male, and 21 patients were Japanese. The median time from COVID-19 symptom onset to remdesivir administration was 5 days (range, 1–9 days). The median time from remdesivir administration to the elevation of pancreatic enzyme levels was 5 (range: 1–10) days; 16 patients received pulsed steroid therapy, 6 received non-pulse steroid therapy, and one patient did not receive steroids. The median duration of steroid administration was 12.5 (range: 5–69) days. Among the 23 patients, eight were regular drinkers, two were occasional drinkers, and six were non-drinkers, and the drinking status of seven patients was unknown. Of the 23 patients, amylase, lipase, and both were elevated more than three times the upper limit of the normal range in 6, 21, and one patient, respectively. In addition, six of the 23 patients had complications of abdominal pain; one among these patients reported nausea. Computed tomography (CT) was performed in the 6 patients after the increase in pancreatic enzyme levels and complaints of abdominal pain and nausea, and acute pancreatitis was confirmed in three patients (Fig. 2). No signs of pancreatitis were observed in the CT scans of the remaining three patients. Gallstones were found on CT scans in five of the 23 patients, where all five had no history of cirrhosis. Seven of the 23 patients were positive for either or both hepatitis B surface antibody (HBsAb) and hepatitis B core antibody (HBcAb). However, no hepatitis B surface antigen (HBsAg) was detected in any of the cases. Finally, seven of the 23 patients were diagnosed with acute pancreatitis according to the 2012 revised Atlanta Classification^10^.

**Table 3:** Characteristics of COVID-19 patients with elevated pancreatic enzyme levels during remdesivir treatment.

Patients characteristics (N = 23)	N	(%)
Age, years, median (range)	73 (50–91)	
**Sex**		
Male	14	60.9
Female	9	39.1
**Race**		
Japanese	21	91.3
East-Asian	2	8.7
**Smoking history**		
Never smoker	10	43.5
Ex-smoker	4	17.4
Current smoker	5	21.7
Unknown	4	17.4
**History of alcohol consumption**		
Regular drinking	8	34.8
Drinking on occasion	2	8.7
Never	6	26.1
Unknown	7	30.4
**Comorbidities**		
Diabetes mellitus	10	43.5
Hypertension	13	56.5
Hyperlipidemia	5	21.7
**Severity of illness categories by NIH classification**		
Severe	8	34.8
Critical	15	65.2
**Duration of remdesivir administration, days**		
2	2	8.7
5	17	73.9
7	1	4.3
10	3	13.0
Duration from COVID-19 onset to remdesivir administration, days, median (range)	5 (1–9)	
**Steroid dose**		
Steroid pulse	16	69.6
Non-pulse	6	26.1
No	1	4.3
Duration of steroid administration, days, median (range)	12.5 (5–69)	
**Outcome**		
Discharged	20	88.1
Death	2	8.7
Hospitalized	1	4.3
Duration of hospitalization, days, median, (range)	16 (4–65)

### Hospitalized COVID-19 patients without remdesivir treatment

A total of 159 patients whose hospitalizations were between February 5, 2020 and January 31, 2021 were enrolled in this study. The baseline characteristics of the patients are presented in Table 4. Based on the COVID-19 severity classification according to the NIH criteria, four cases were asymptomatic, 22 were mild, 60 were moderate, 48 were severe, and 25 were critical. Eighteen patients received pulse steroid therapy, 56 received non-pulse steroid therapy, and 85 did not receive steroid therapy at all. Increased pancreatic enzyme levels were observed in three of the 159 patients. CT was performed in one of the three patients after the increase in pancreatic enzyme levels, but no signs of pancreatitis were observed on CT scans.

**Table 4:** Characteristics of COVID-19 patients who did not receive remdesivir.

Patients characteristics (N = 159)	N	(%)
Age, years, median (range)	71 (23–94)	
**Sex**		
Male	82	51.6
Female	77	48.4
**Race**		
Japanese	140	88.1
East-Asian	4	2.5
South-Asian	6	3.8
Lationo	1	0.6
Caucasian	7	4.4
Mix	1	0.6
**Severity of illness categories by NIH classification**		
Asymptomatic	4	2.5
Mild	22	13.8
Moderate	60	37.7
Severe	48	30.2
Critical	25	15.7
**Steroid dose**		
Steroid pulse	18	11.3
Non-pulse	56	35.2
No	85	53.5
**Comorbidities**		
Diabetes mellitus	44	28.0
Hypertension	47	29.9
Hyperlipidemia	28	17.8
**D-dimer, µg/mL**		
Normal	34	21.7
Elevated (> 1.0 μg/mL)	112	71.3
Unexamined	13	8.3
**Increase in pancreatic enzyme levels**		
Grade 3	3	1.9
Grade 4	0	0
**Outcome**		
Discharged	143	89.9
Death	15	9.4
Hospitalized	1	0.6

Baseline demographic and clinical characteristics of 157 patients who did not received remdesivir between February 5, 2020 and January 31, 2021.

### Results of the statistical analysis

The associations between acute pancreatitis and grade 3 or higher pancreatic enzyme level elevation and patient characteristics are shown in Table 5. The proportion of patients with elevated pancreatic enzyme levels was predominantly higher among remdesivir-treated patients (*p* = 0.001), steroid-treated patients (*p* = 0.008), and patients with NIH criteria of critical COVID-19 (*p* = 0.000). Logistic regression analysis was performed using the onset of acute pancreatitis and increase in pancreatic enzyme levels above grade 3 severity as dependent variables and whether remdesivir was administered, whether steroids were administered, sex, age (< 65 years or ≥ 65 years), whether patients had diabetes, hypertension, hyperlipidemia, and elevated D-dimer levels, as well as the NIH disease severity criteria for COVID-19 (critical vs. not critical) as covariates. Participants meeting the NIH criteria for critical COVID-19 and receiving remdesivir had a higher risk of acute pancreatitis and a grade 3 or higher increase in pancreatic enzyme levels, with an adjusted OR of 10.137 (95% CI: 3.631 to 28.299, *p* = 0.000) for the NIH criteria and 4.565 (95% CI: 1.113–18.724, *p* = 0.035) for remdesivir administration. Additionally, administration of steroids, sex, age, diabetes, hypertension, hyperlipidemia, and elevated D-dimer levels did not have a high risk of acute pancreatitis and grade 3 or higher increase in pancreatic enzyme levels (Table 6).

**Table 5:** Increase in pancreatic enzyme levels and patient characteristics.

	Total	Elevated pancreatic enzymes ( +)	Elevated pancreatic enzymes ( −)	*p* value
	N = 360	N = 26	N = 334	
**Remdesivir**				
+	201	23 (11.4%)	178 (88.5%)	0.001*
−	159	3 (1.9%)	156 (98.1)	
**Sex**				
Male	208	16 (7.7%)	192 (92.3%)	0.687
Female	152	10 (6.6%)	142 (93.4%)	
**Age, years**				
< 65	144	9 (6.3%)	135 (93.8%)	0.561
≥ 65	216	17 (7.9%)	199 (92.1)	
**Steroid use**				
+	267	25 (9.4%)	242 (90.6)	0.008*
−	93	1 (1.1%)	92 (98.9%)	
**Diabetes**				
+	111	10 (9.0%)	101 (91.0%)	0.382
−	249	16 (6.4%)	233 (93.6%)	
**Hypertension**				
+	156	15 (9.6%)	141 (90.4%)	0.125
−	204	11 (5.4%)	193 (94.6%)	
**Hyperlipidemia**				
+	68	5 (7.4%)	63 (92.6%)	0.963
−	292	21 (7.2%)	271 (92.8%)	
**D-dimer**				
Elevated	284	22 (7.7%)	262 (92.3%)	0.458
Normal or NE	76	4 (5.4%)	72 (94.7%)	
**NIH**				
Critical	83	18 (21.7%)	65 (78.3%)	0.000*
Not critical	277	8 (2.9%)	269 (97.1%)	

The association between a Grade 3 or higher increase in pancreatic enzyme levels or acute pancreatitis and patient characteristics was analyzed using the chi-square test.

*NE* Not examined, *NIH* The National Institutes of Health.

*Statistically significant (*p* ≤ 0.05).

**Table 6:** Results of univariate and multivariate analyses of increased pancreatic enzyme levels in COVID-19 patients.

Cutoff		Univariate analysis		Multivariate analysis	
		HR (95% CI)	*p*	HR (95% CI)	*p*
Remdesivir	(+) versus (−)	6.719 (1.979–22.808)	0.002*	4.565 (1.113–18.724)	0.035*
Steroid use	(+)versus (−)	9.504 (1.269–71.156)	0.028*	2.433 (0.238–24.911)	0.454
Age, years	< 65 versus ≥ 65	1.281 (0.555–2.959)	0.561	0.498 (0.174–1.423)	0.193
Sex	Male versus female	0.845 (0.372–1.917)	0.687	1.235 (0.493–3.094)	0.653
Diabetes	(+)versus (−)	1.442 (0.633–3.286)	0.384	1.291 (0.491–3.398)	0.605
Hypertension	(+)versus (−)	1.867 (0.832–4.186)	0.13	0.954 (0.361–2.525)	0.925
Hyperlipidemia	(+)versus (−)	1.024 (0.372–2.821)	0.963	0.761 (0.237–2.447)	0.647
D-dimer	Elevated or not	1.511 (0.505–4.526)	0.46	0.554 (0.145–2.125)	0.389
NIH	Critical versus not critical	9.312 (3.879–22.354)	0.000*	10.137 (3.631–28.299)	0.000*

The logistic regression model was used to calculate the adjusted odds ratio (OR) with 95% confidence interval (CI) for risks of increase in Grade 3 or higher pancreatic enzyme levels or onset of acute pancreatitis.

*Statistically significant (*p* ≤ 0.05).

## Discussion

This retrospective analysis of COVID-19 patients treated with remdesivir at our institution indicated that increased risk of acute pancreatitis and grade 3 or higher increase in pancreatic enzyme levels is associated with remdesivir administration. The results of this study differed from those of previously published studies. To the best of our knowledge, this is the first study to report a high incidence of acute pancreatitis or a severe increase in pancreatic enzyme levels in COVID-19 patients treated with remdesivir. In the ACTT-1 study, 538 patients received remdesivir, but acute pancreatitis was not reported^4,5^. Similarly, in two other studies where 384 and 155 patients received remdesivir, pancreatitis was not reported^6,7^. Only one case of acute pancreatitis was reported in the ACTT-2 study in the remdesivir plus baricitinib group^8^.

However, compared to ACTT-1 and other studies, most patients in our study were treated with steroids, which increased the incidence of acute pancreatitis approximately two-fold^11^. Thus, steroids may have contributed to the development of pancreatitis in our patients. However, pancreatitis was not reported in the RECOVERY trial, which was the first to demonstrate the effects of dexamethasone on COVID-19^12^.

As for the risk of pancreatitis, gallstones were observed on the CT scans of 5 of 23 patients, but none of them had findings suggestive of gallstone pancreatitis. Regarding the history of liver disease, there were no patients with cirrhosis, and only seven patients had previous hepatitis B virus (HBV) infections. There was little difference in alcohol consumption history between the elevated pancreatic enzyme group and the remdesivir group as a whole, and this percentage was similar to that observed in The National Health and Nutrition Survey in Japan, 2019 conducted by the Ministry of Health, Labour and Welfare^13^. Other etiological causes of acute pancreatitis were excluded from the patients diagnosed with acute pancreatitis.

Preclinical data on the use of GS-5734 (remdesivir) for rhesus monkeys infected with the Ebola virus showed increased serum lipase levels several days after administration, suggesting that remdesivir leads to an increased production of pancreatic enzymes^14^. A literature search also revealed multiple reports of increased pancreatic enzyme levels during the course of COVID-19^15–17^.

Therefore, we reviewed the data of hospitalized COVID-19 patients who did not receive remdesivir at our institution. A total of 159 patients with COVID-19 hospitalized between February 2020 and January 2021 were enrolled. Increased pancreatic enzyme levels were observed in 3 of the 159 patients (Table 4). Logistic regression analysis was performed. Participants meeting the NIH criteria for critical COVID-19 and who received remdesivir had a higher risk of acute pancreatitis and grade 3 or higher increase in pancreatic enzyme levels, with an adjusted OR of 10.137 (95% CI: 3.631 to 28.299, *p* = 0.000) for the NIH criteria and 4.565 (95% CI: 1.113–18.724, *p* = 0.035) for remdesivir administration.

The strength of our study is that these data are from a designated medical institution for Class I Infectious Diseases (e.g., Ebola hemorrhagic fever), and all COVID-19 patients were treated by infectious disease specialists or respiratory specialists as attending physicians or supervisors, which enabled us to provide advanced medical care. Moreover, these findings were obtained from patients with COVID-19 treated at the same institution and using the same clinical decision. When a large number of medical institutions are involved, it is difficult to ensure homogeneity of management, and thus, data quality, and the evaluation of disease severity and adverse events is likely to vary.

This study has some limitations. First, this was a small, uncontrolled, single-center, retrospective study. Second, most patients who received remdesivir were Japanese, and the possibility of racial diversity could not be ruled out. However, there was no elevation in pancreatic enzyme levels in a study among ethnically related Chinese participants^6^ or in a global study that included a proportion of Asians. These results lead us to believe that the increased risk of pancreatitis is not only present in patients of Asian descent. Therefore, pancreatitis or increase in pancreatic enzyme levels may have been incorrectly judged as a manifestation of COVID-19. Third, our analysis is limited by a lack of data on comorbidity and obesity, which are risk factors for hospitalization with COVID-19, except for diabetes, hypertension, and hyperlipidemia, which could be assessed based on glycated hemoglobin levels and regular medication prescriptions. Fourth, in the present study, CT was performed in only six out of 23 cases, radiologically confirmed in three cases (50%), and acute pancreatitis was confirmed in three cases. Therefore, elevated pancreatic enzymes may be reactive and not clinically relevant. Finally, as many COVID-19 patients treated with remdesivir at our hospital had increased pancreatic enzyme levels, there were actually several cases wherein remdesivir administration was discontinued early when pancreatic enzyme levels were elevated to grade 2, or cases where nafamostat or ulinastatin were co-administered when the pancreatic enzyme levels were increased in patients whose cycle threshold values of SARS-COV-2 reverse transcription polymerase chain reaction were low and remdesivir administration was continued; thus, the increase in pancreatic enzyme levels could have been underestimated.

It is unclear whether pancreatic enzyme levels are elevated in severe COVID-19 infection itself as part of multiorgan failure, or in conjunction with unknown factors. Our results suggest that remdesivir administration was an independent predictor of increased pancreatic enzyme levels, in addition to COVID-19 severity. Follow-up studies on remdesivir administration and elevated pancreatic enzyme levels are warranted.

In conclusion, in the present study, 23 of 201 patients treated with remdesivir had elevated pancreatic enzyme levels. Additionally, logistic regression analysis revealed remdesivir administration as an independent predictor of increased pancreatic enzyme levels. Therefore, we propose monitoring pancreatic enzyme levels in COVID-19 patients to detect the onset of pancreatitis during the course of remdesivir treatment for COVID-19.
